# Supplementary material for: Identification of Freezing-Responsive microRNAs and Their Targets in Chinese Jujube by Small RNA and Degradome Sequencing
Source: Int J Mol Sci. 2026 Sep 4;27(17):7912. doi: 10.3390/ijms27177912 (PMC13566890; doi:10.3390/ijms27177912)
Supplement: Supplementary file 1 [file ijms-27-07912-s001.zip › Supplementary Material S2/novel_miR_10.pdf]

Provisional ID	:	novel_miR_10
Score total	:	1262.7
Score for star read(s)	:	3.9
Score for read counts	:	1260.9
Score for mfe	:	0.1
Score for randfold	:	-2.2
Score for cons. seed	:	
Total read count	:	2485
Mature read count	:	2483
Loop read count	:	0
Star read count	:	2

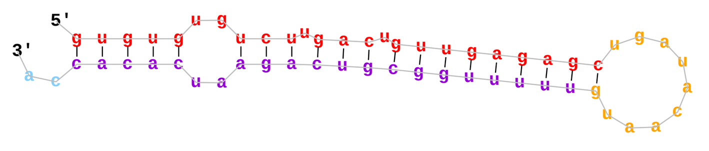

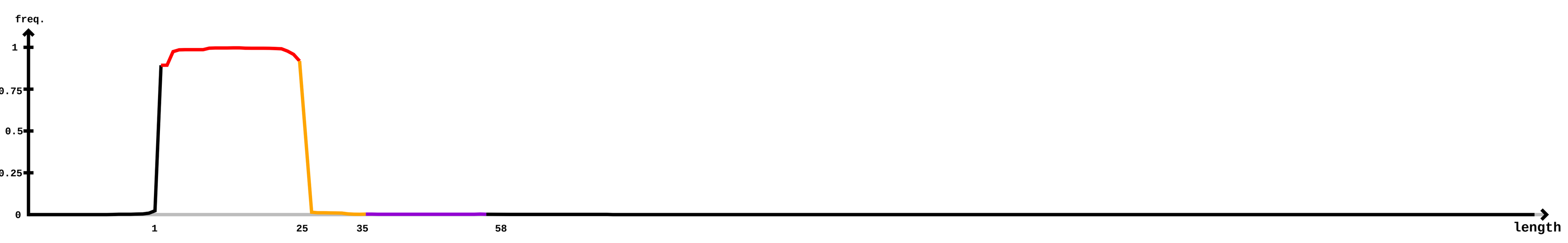

Mature Star

```
5'- ucuacugcucaaugagaauugugugugucuugacuguugagagcugauacaauguuuuuggcgucagaaucacaccauauuuuuugccagccuuccucuugagcuaaaauuaauccuugcauaucacauaaauaugcauuugcuuuugggguuccucauaugccauaccaugacaauaaccucugugccuuucuuuuuauaaaaaauccaugcaaagaugcccuuaagcuuuauaggaaaagucccaccaaca -3'   obs
    ucuacugcucaaugagaauugugugugucuugacuguugagagcugauacaauguuuuuggcgucagaaucacaccauauuuuuugccagccuuccucuugagcuaaaauuaauccuugcauaucacauaaauaugcauuugcuuuugggguuccucauaugccauaccaugacaauaaccucugugccuuucuuuuuauaaaaaauccaugcaaagaugcccuuaagcuuuauaggaaaagucccaccaaca        exp
    ......(((....((((((.(((((..(((.(((.(((((((((.........)))))))))))))))..)))))...))))))....))).(((((...(((((......(((..(((((((......)))))))(((((...((((((...)))))))(((((...((((((....(((...........(((........)))..........)))...)))))).))))))))......)))))...)))))..............   reads   mm   sample
    ............ugagaauugugugugucuugac..........................................   2   0   LT-2
    .............gagaauugugugugucuugac..........................................   3   0   LT-1
    ................aCuugugugugucuugacu.........................................   1   1   LT-3
    ................aauugugugugucuugacuguuU.....................................   2   1   JCK-2
    .................auugugugugucuugacuguu......................................   1   0   JCK-2
    .................auugugugugucuugacuguugag...................................   1   0   LT-1
    ..................uugugugugucuugacuguug.....................................   1   0   LT-3
    ..................uugugugugucuugacuguugag...................................   1   0   LT-3
    ..................uugugugugucuugacuguugaga..................................   5   0   JCK-3
    ..................uugugugugucuugacuguugaga..................................   3   0   JCK-1
    ..................uugugugugucuugacuguugaga..................................   2   0   JCK-2
    ...................ugugugugucuugacuguuga....................................   1   0   LT-1
    ...................ugugugugucuugacuguuga....................................   1   0   LCK-3
    ...................ugugugugucuugacuguuUag...................................   1   1   JCK-1
    ...................ugugugugucuugacuguugag...................................   4   0   LCK-3
    ...................ugugugugucuugacuguugag...................................   1   0   JCK-3
    ...................ugugugugucuugacuguugag...................................   3   0   JCK-1
    ...................ugugugugucuugacuguugag...................................   1   0   JCK-2
    ...................ugugugugucuugacuguuUag...................................   3   1   JCK-3
    ...................ugugugugucuugacuguugaga..................................   3   0   LCK-3
    ...................ugugugugucuugacuguugagag.................................   2   0   JCK-2
    ...................ugugugugucuugacuguugagag.................................   1   0   JCK-1
    ...................ugugugugucuugacuguugagag.................................   2   0   JCK-3
    ...................ugugugugucuugacuguugaAag.................................   1   1   LT-1
    ...................ugugugugucuugacuguugagag.................................   4   0   LT-1
    ...................ugugugugucuugacuguugagag.................................   2   0   LT-3
    ...................ugugugugucuugacuguuUagag.................................   2   1   JCK-3
    ...................ugugugugucuugacuguugagagc................................   1   0   JCK-2
    ...................ugugugugucuugacuguugagagc................................   1   0   JCK-1
    ...................Cgugugugucuugacuguugagagc................................   1   1   LT-3
    ...................ugugugugucuugacuguuUagagc................................   1   1   JCK-3
    ....................gugugugucuugacuguuga....................................   1   0   LT-1
    ....................gugugugucuugacuguugag...................................   2   0   LT-2
```

```
                    Mature                  Star
ucuacugcucaaugagaauugugugugucuugacuguugagagcugauacaauguuuuuggcgucagaaucacaccauauuuuuugccagccuuccucuugagcuaaaauuaauccuugcauaucacauaauaugcauuugcuuuuggguuccucauaugccauaccaugacaauaaccucugugccuuucuuuuuauaaaaauccaugcaaagaugcccuuaagcuuuauaggaaaagucccaccaaca
....................gugugugucuugacuguugag        2     0    JCK-1
....................gugugugucuugacuguugag        3     0    LT-3
....................gugugugucuugacuguugag        6     0    LCK-3
....................gugugugucuugacuguuUag        1     1    JCK-1
....................gugugugucuugacuguuUag        3     1    JCK-2
....................gugugugucuugacuguugaga       2     0    JCK-3
....................gugugugucuugacuguugaga       12    0    LCK-2
....................gugugugucuugacuguugaga       3     0    LT-1
....................gugugugucuugacuguugaga       14    0    LCK-1
....................gugugugucuugacuguuUaga       2     1    LT-1
....................gugugugucuugacuguugagag      1     0    LT-3
....................gugugugucuugacuguugaAag      1     1    LT-3
....................gugugugucuugacuguuUagag      1     1    JCK-1
....................gugugugucuugacuguuUagag      1     1    LT-3
....................gugugugucuugacuguugagag      2     0    LCK-3
....................gugugugucuugacuguuUagag      1     1    JCK-3
....................gugugugucuugacuguuUagag      3     1    LT-2
....................gugugugucuugacuguugagag      2     0    LT-1
....................Uugugugucuugacuguugagagc     1     1    LCK-1
....................gugugugucuugacuguugaAagc     12    1    LT-3
....................gugugugucuugacuguugagagc     283   0    LT-1
....................gugugugucuugacuguuUagagc     16    1    LT-3
....................gugugugucuugacuguugagagc     208   0    JCK-2
....................gugugugucuugacuguuUagagc     38    1    JT-1
....................gugugugucuugacuguugagagc     28    0    JT-2
....................gugugugucuugacuguugagagc     146   0    LT-2
....................gugugugucuugCcuguugagagc     1     1    JCK-1
....................gugugugucuugacuguugagagc     269   0    LT-3
....................gugugugucuugacuguugaAagc     16    1    LCK-3
....................guAugugucuugacuguugagagc     1     1    LCK-3
....................Augugugucuugacuguugagagc     3     1    JCK-2
....................gugugugucuugacuguugUgagc     1     1    JCK-2
....................gugugugucuugacuguugagagc     136   0    JCK-3
....................Augugugucuugacuguugagagc     1     1    LT-2
....................gugugugucuugaAuguugagagc     1     1    LCK-3
....................gugGgugucuugacuguugagagc     2     1    LT-3
....................gugugugucuugacuguuUagagc     71    1    JCK-1
....................gugugugucuugacuguuUagagc     95    1    JCK-3
....................gugugugucuugacuguugagagc     177   0    LCK-3
....................gugugugucuugacuguugagagU     1     1    LT-2
....................gugugugucuugacuguugagCgc     1     1    LT-2
....................gugugugucuugacuguugagagc     124   0    JT-1
....................gugugugucuugacuguCgagagc     1     1    LT-3
....................gugugugucuugacuguugNgagc     1     1    JCK-1
....................gugugugucuugacuguugagagc     53    0    LCK-1
....................gugugugucuugaAuguugagagc     1     1    LT-3
....................Augugugucuugacuguugagagc     1     1    LT-1
....................Uugugugucuugacuguugagagc     1     1    LT-1
....................gugugugucuugacuguugagagc     34    0    JT-3
....................gugugugucuugacuguugagagc     133   0    JCK-1
....................gugugugucuugacuguugaAagc     9     1    LT-2
....................gugugugucuugacuguugagagc     58    0    LCK-2
....................gugugugucuugacuguugaAagc     15    1    LT-1
....................gugugugucuugacuguugCgagc     1     1    JCK-2
....................gugugugucuugacuguuUagagc     13    1    LT-1
....................gugugugucuugacuguuUagagc     8     1    JT-3
....................gugugugucuugacuguuUagagc     12    1    LT-2
....................gugugugucuugacuguuUagagc     5     1    LCK-3
....................gugugugucuugacuguuUagagc     4     1    JT-2
....................gugugugucuugacuguugagagU     1     1    LT-3
....................gugugugucuugaAuguugagagc     3     1    LT-2
....................gugugugucuugacuguuUagagc     147   1    JCK-2
....................Augugugucuugacuguugagagc     1     1    LCK-3
.....................ugugugucuugacuguugagagc     1     0    JCK-2
......................gugugucuugacuguuga         1     0    LCK-3
......................gugugucuugacuguuUag        3     1    JCK-2
......................gugugucuugacuguugaga       1     0    LT-3
......................gugugucuugacuguugaga       2     0    JCK-2
......................gugugucuugacuguugagag      1     0    LT-1
......................Cugugucuugacuguugagag      1     1    LT-3
```

```
                  Mature                Star
ucuacugcucaaugagaauugugugugucuugacuguugagagcugauacaauguuuuuggcgucagaaucacaccauauuuuuugccagccuuccucuugagcuaaaauuaauccuugcauaucacauaauaugcauuugcuuuuggguuccucauaugccauaccaugacaauaaccucugugccuuucuuuuuauaaaaauccaugcaaagaugcccuuaagcuuuauaggaaaagucccaccaaca
......................gugugucuugacuguugagag...............................................................................................................................................................................   39   0   LCK-1
......................gugugucuugacuguugagag...............................................................................................................................................................................   31   0   LCK-2
......................gugugucuugacugGugagag...............................................................................................................................................................................   1    1   LCK-1
......................gugugucuugacuguugagagc..............................................................................................................................................................................   17   0   JCK-3
......................gugugucuugacuguugaAagc..............................................................................................................................................................................   5    1   LCK-3
......................gugugucuugacuguuUagagc..............................................................................................................................................................................   1    1   LT-1
......................gugugucuugacuguugagagc..............................................................................................................................................................................   11   0   JCK-1
......................gGgugucuugacuguugagagc..............................................................................................................................................................................   1    1   LT-1
......................gugugucuugacuguugagagc..............................................................................................................................................................................   6    0   LT-2
......................gugugucuugacuguugagagc..............................................................................................................................................................................   11   0   LT-3
......................gugugucuugacuguugagagc..............................................................................................................................................................................   19   0   JCK-2
......................gugugucuugacuguuUagagc..............................................................................................................................................................................   5    1   JCK-1
......................gugugucuugacuguuUagagc..............................................................................................................................................................................   7    1   JCK-2
......................gugugucuugacuguugagagc..............................................................................................................................................................................   4    0   LCK-3
......................gGgugucuugacuguugagagc..............................................................................................................................................................................   1    1   LCK-3
......................gugugucuugacuguuUagagc..............................................................................................................................................................................   5    1   JCK-3
......................gugugucuugacuguugagagc..............................................................................................................................................................................   14   0   LT-1
......................gugugucuugacuguuUagagcu.............................................................................................................................................................................   1    1   JCK-3
......................gugugucuugacuguugagagcu.............................................................................................................................................................................   2    0   LT-3
......................gugugucuugacuguuUagagcu.............................................................................................................................................................................   1    1   JCK-1
......................gugugucuugacuguugagagcu.............................................................................................................................................................................   5    0   LT-2
......................gugugucuugacuguugaAagcu.............................................................................................................................................................................   1    1   LT-1
......................gugugucuugacuguugaAagcu.............................................................................................................................................................................   1    1   LT-3
......................gugugucuugacuguugagagcug............................................................................................................................................................................   1    0   LT-3
......................gugugucuugacuguuUagagcug............................................................................................................................................................................   2    1   JCK-2
......................gugugucuugacuguuUagagcug............................................................................................................................................................................   1    1   JCK-1
......................gugugucuugacuguugagagcug............................................................................................................................................................................   3    0   LT-1
.......................ugugucuugacuguugagagc..............................................................................................................................................................................   2    0   LT-1
.......................ugugucuugacuguugagagc..............................................................................................................................................................................   2    0   JCK-3
.......................ugugucuugacuguugaAagc..............................................................................................................................................................................   2    1   LT-1
.......................ugugucuugacuguugagagc..............................................................................................................................................................................   2    0   JCK-1
.......................ugugucuugacuguugagagc..............................................................................................................................................................................   5    0   LT-3
.......................ugugucuugacuguugagagc..............................................................................................................................................................................   2    0   LT-2
.......................ugugucuugacuguugagagc..............................................................................................................................................................................   8    0   JCK-2
.......................ugugucuugacuguugaAagc..............................................................................................................................................................................   2    1   LT-2
.......................ugugucuugacuguugagagcu.............................................................................................................................................................................   1    0   LT-3
.......................ugugucuugacuguugagagcu.............................................................................................................................................................................   1    0   JCK-1
........................gugucuugacuguugaAag...............................................................................................................................................................................   1    1   LT-3
........................gugucuugacuguugagagcug............................................................................................................................................................................   1    0   LT-1
........................gugucuugacuguuUagagcugau..........................................................................................................................................................................   1    1   JCK-3
............................cuugacuguugagagcugau..........................................................................................................................................................................   1    0   JCK-2
............................cuugacuguugagagcugauac........................................................................................................................................................................   2    0   LT-2
............................cuugCcuguugagagcugauaca.......................................................................................................................................................................   1    1   LT-3
............................cuugacuguugagagcugauaca.......................................................................................................................................................................   4    0   LT-1
............................cuugacuguuUagagcugauaca.......................................................................................................................................................................   1    1   LT-3
............................cuugacuguugagagcugauaca.......................................................................................................................................................................   2    0   LT-2
............................cuugacuguugagagcugauaca.......................................................................................................................................................................   1    0   JCK-2
............................cuugacuguugagagcugauaca.......................................................................................................................................................................   2    0   JCK-1
............................cuugacuguuUagagcugauaca.......................................................................................................................................................................   3    1   JCK-3
............................cuugacuguugagagcugauacaC......................................................................................................................................................................   2    1   LCK-3
............................cuugacuguugagagcugauacaC......................................................................................................................................................................   1    1   LT-3
............................cuugacuguugagagcugauacaC......................................................................................................................................................................   2    1   LT-1
.............................uugacuguugagagcugaua.........................................................................................................................................................................   1    0   JCK-2
.............................uugacuguugagagcugauaca.......................................................................................................................................................................   1    0   LT-3
.............................uugacuguuUagagcugauacaau.....................................................................................................................................................................   1    1   LT-1
................................acuguuUagagcugauacaauguu..................................................................................................................................................................   1    1   JCK-2
................................acuguuUagagcugauacaauguu..................................................................................................................................................................   1    1   JCK-1
...................................................auguuuuuggcgucagaaucacG................................................................................................................................................................   2    1   JCK-2
...................................................auguuuuuggcgucagaaucacG................................................................................................................................................................   1    1   JCK-3
......................................................uuuuuggcgucagaaucacac...............................................................................................................................................................   1    0   JCK-3
......................................................uuuuuggcgucagaaucacac...............................................................................................................................................................   1    0   JCK-2
.........................................................................accauauuuuuugccaAccuuc...........................................................................................................................................................   1    1   JCK-3
.........................................................................accauauuuuuugccaAccuuc...........................................................................................................................................................   2    1   JCK-2
```
